# Tartary Buckwheat Peptides Prevent Oxidative Damage in Differentiated SOL8 Cells via a Mitochondria-Mediated Apoptosis Pathway

**DOI:** 10.3390/nu17132204

**Published:** 2025-07-02

**Authors:** Yifan Xu, Yawen Wang, Min Yang, Pengxiang Yuan, Weikang Xu, Tong Jiang, Jian Huang

**Affiliations:** 1 National Institute for Nutrition and Health, Chinese Center for Disease Control and Prevention, Beijing 100050, China; xuyifanuk2020@163.com (Y.X.); huangjian@ninh.chinacdc.cn (J.H.); 2 Key Laboratory of Public Nutrition and Health, National Health Commission of the People’s Republic of China, Beijing 100050, China; 3 Air Force Medical Center of PLA, Air Force Medical University, Beijing 100142, China; yawen0723@163.com; 4 School of Food and Pharmacy, Zhejiang Ocean University, Zhoushan 316022, China; 2022111@zjou.edu.cn (M.Y.); yuan_pengxiang@163.com (P.Y.); xuweikang1015@163.com (W.X.)

**Keywords:** Tartary buckwheat peptides, oxidative damage, SOL8 cells, mitochondria, ROS

## Abstract

**Background:** Under oxidative stress conditions, the increased levels of reactive oxygen species (ROS) within cells disrupt the intracellular homeostasis. Tartary buckwheat peptides exert their effects by scavenging oxidative free radicals, such as superoxide anion and hydrogen peroxide, thereby reducing oxidative damage within cells. Meanwhile, these peptides safeguard mitochondria by maintaining the mitochondrial membrane potential, decreasing the production of mitochondrial oxygen free radicals, and regulating mitochondrial biogenesis and autophagy to preserve mitochondrial homeostasis. Through these mechanisms, Tartary buckwheat peptides restore the intracellular redox balance, sustain cellular energy metabolism and biosynthesis, and ensure normal cellular physiological functions, which is of great significance for cell survival and adaptation under oxidative stress conditions. **Objectives:** In this experiment, a classical cellular oxidative stress model was established. Indicators related to antioxidant capacity and mitochondrial membrane potential changes, as well as pathways associated with oxidative stress, were selected for detection. The aim was to elucidate the effects of Tartary buckwheat oligopeptides on the metabolism of cells in response to oxidative stress. **Methods:** In this study, we established an oxidative damage model of mouse skeletal muscle myoblast (SOL8) cells using hydrogen peroxide (H_2_O_2_), investigated the pre-protective effects of Tartary buckwheat oligopeptides on H_2_O_2_-induced oxidative stress damage in SOL8 cells at the cellular level, and explored the possible mechanisms. The CCK-8 method is a colorimetric assay based on WST-8-[2-(2-methoxy-4-nitrophenyl)-3-(4-nitrophenyl)-5-(2,4-disulfophenyl)-2H-tetrazolium, monosodiumsalt], which is used to detect cell proliferation and cytotoxicity. **Results:** The value of CCK-8 showed that, when the cells were exposed to 0.01 mmol/L H_2_O_2_ for 1 h and 10 mg/mL Tartary buckwheat oligopeptides intervention for 48 h, these were the optimal conditions. Compared with the H_2_O_2_ group, the intervention group (KB/H_2_O_2_ group) showed that the production of ROS was significantly reduced (*p* < 0.001), the malondialdehyde (MDA) content was significantly decreased (*p* < 0.05), and the activity of catalase (CAT) was significantly increased (*p* < 0.01); the mitochondrial membrane potential in the KB/H_2_O_2_ group tended to return to the level of the control group, and they all showed dose-dependent effects. Compared with the H_2_O_2_ group, the mRNA expression of KEAP1 in the KB/H_2_O_2_ group decreased, while the mRNA expression of NRF2α, HO-1, nrf1, PGC-1, P62, and PINK increased. **Conclusions**: Therefore, Tartary buckwheat oligopeptides have a significant pre-protective effect on H_2_O_2_-induced SOL8 cells, possibly by enhancing the activity of superoxide dismutase, reducing ROS attack, balancing mitochondrial membrane potential, and maintaining intracellular homeostasis.

## 1. Introduction

Skeletal muscle is one of the major metabolic tissues in the human body. Skeletal muscle myoblasts (SOL8) contain a large number of mitochondria, which are the central organelles for cellular energy metabolism and also the primary targets for the toxic effects of many environmental chemical xenobiotics [1]. The mitochondrial respiratory chain has been widely confirmed to be the main source of reactive oxygen species (ROS) production. Excessive superoxides, if not cleared by endogenous antioxidants or related enzymes, can lead to mitochondrial oxidative stress damage and functional disorders, thereby triggering a variety of pathological changes, including cell death [2]. The respiratory chain of mitochondria generates the vast majority of ROS in the body. There exists an antioxidant system against ROS in the respiratory chain, such as coenzyme Q (CoQ10) and cytochrome C. Together with other antioxidants in the body, such as superoxide dismutase (SOD) and catalase (CAT), they form an antioxidant system to eliminate ROS produced during the transfer of the respiratory chain. Therefore, under normal physiological conditions, the body’s oxidation and antioxidant systems are in balance with each other [3].

Irregular, prolonged, and high-intensity vigorous exercise can disrupt the balance between the oxidation and antioxidant systems, thereby causing oxidative damage to the body and inducing mitochondrial dysfunction. Since the ROS produced by cells exceeds the clearance capacity of the body’s own antioxidant system, the balance between oxidation and antioxidant action in the body is disrupted [4,5]. Free radicals are highly reactive substances that are continuously produced in the body’s metabolism and can damage the body itself. They can directly or indirectly exert their strong oxidizing effects, thereby damaging macromolecules and various cellular components in the body. The production and elimination of free radicals in the body are in a dynamic equilibrium. When the production of free radicals is excessive or the elimination is reduced, it will lead to the accumulation of free radicals, thereby causing oxidative damage [6,7]. Hydrogen peroxide is a very unstable oxide that can decompose to produce hydroxyl radicals, leading to cell peroxidation damage. It is the substance most commonly used to establish in vitro oxidative stress models at present.

Exogenous antioxidant nutrients are the most powerful “umbrella” for maintaining normal mitochondrial function and can effectively protect the integrity of mitochondrial function and delay the decline of mitochondrial function. Tartary buckwheat oligopeptides have the advantages of fast absorption, low energy consumption, and non-saturated carriers. They have strong activity and biological diversity and can comprehensively regulate human physiological functions. Moreover, functional zones may generally exist in the peptide chains of nutritional proteins. These low-molecular-weight peptides obtained by appropriate protease hydrolysis may be released, thereby obtaining active peptides with multiple functions. In addition, Tartary buckwheat protein has been proven to have strong active peptide components that can scavenge superoxide anions [8]. Modern nutritional studies believe that, when the body is in a state of fatigue, the intake of bioactive peptides will effectively supplement active substances and nutrients, improve cell metabolism, restore the disturbed internal environment, and enable the body’s various systems and organs to work normally, thereby achieving the goal of delaying or eliminating fatigue. Oligopeptides, as an important raw material for functional foods, are favored by people engaged in physical, mental, and sports activities [9].

In order to further explore the effects of Tartary buckwheat oligopeptides on skeletal muscle oxidative damage at the cellular level, this experiment will use in vitro cultured SOL8 mouse skeletal muscle myoblasts as research materials and select H_2_O_2_-induced SOL8 oxidative damage models to explore the antioxidant damage effects of Tartary buckwheat oligopeptides on SOL8 myoblasts and their possible mechanisms in terms of cell survival rate, lactate dehydrogenase (LDH) and catalase (CAT) activity in cells, and the content of malondialdehyde (MDA) and reactive oxygen species (ROS) in cells.

In skeletal muscle cells, mitochondria are the core organelles of cellular energy metabolism, responsible for producing most of the energy (ATP) required by cells. Mitochondria play a key role in cellular oxidative stress, especially in skeletal muscle cells, because these cells have high energy demands, and mitochondria produce a large amount of ROS during energy metabolism [10]. Under normal physiological conditions, the level of ROS produced by mitochondria is low, and cells can clear these ROS through their own antioxidant systems (such as SOD and CAT) to maintain the redox balance of cells.

However, when skeletal muscle cells are exposed to oxidative stress, such as in H_2_O_2_-induced oxidative damage models, the level of ROS produced by mitochondria will significantly increase, exceeding the clearance capacity of the cellular antioxidant system. This excessive level of ROS can lead to a decrease in mitochondrial membrane potential and mitochondrial dysfunction, thereby affecting cellular energy metabolism. Damaged mitochondria not only fail to produce ATP normally but also release more ROS, creating a vicious cycle that exacerbates cellular oxidative damage [11]. Under these circumstances, mitophagy is activated as a cellular self-protection mechanism. The PINK1 and Parkin pathways play a key role in recognizing and clearing damaged mitochondria. PINK1 accumulates on the outer membrane of damaged mitochondria, activates Parkin, and then tags damaged mitochondria for autophagic degradation. In addition, the activation of NRF2α also promotes the expression of antioxidant genes (such as HO-1 and NQO1), enhancing the cell’s antioxidant capacity [12]. The upregulation of PGC-1α promotes mitochondrial biogenesis, which helps restore mitochondrial function. Therefore, the relationship between skeletal muscle oxidative damage and mitochondria is mutually influential. Oxidative stress leads to mitochondrial dysfunction, and the recovery and maintenance of mitochondrial function are crucial for alleviating oxidative damage and maintaining cellular homeostasis [13]. By activating mitophagy and enhancing antioxidant defense mechanisms, cells can effectively cope with oxidative stress and reduce oxidative damage to skeletal muscle cells.

This experiment was primarily designed to explore the protective effects of Tartary buckwheat peptides against oxidative stress in sol8 cells. Comparisons were made in several aspects, including intracellular metabolites, changes in mitochondrial membrane potential, and the impact on relevant pathways, in order to demonstrate the ability of Tartary buckwheat oligopeptides to alleviate the level of oxidative stress within cells. We look forward to future experiments, where the effects of bioavailability and in vivo metabolism of the active peptides will be further verified by establishing animal or clinical models.

## 2. Experimental Design and Methods

### 2.1. Cell Culture

SOL8 cells were obtained from the Shanghai Enzyme-linked Biotechnology Company (Shanghai, China). They were cultured in Dulbecco’s modified Eagle medium (DMEM; 12100, Solarbio Co., Beijing, China) supplemented with 10% fetal bovine serum (FBS; S9020, Solarbio Co., Beijing, China), 10,000 units/mL penicillin-streptomycin (P1400, Solarbio Co., Beijing, China), and maintained at 37 °C in a 5% CO_2_. Cells (2 × 10^5^/mL) were harvested and seeded in dishes for 24 h for attachment and differentiation, and then incubated for a week when the cells reached 60% confluency, and maintained in DMEM supplemented with 2% HS (horse serum) and 1% penicillin until treatment.

### 2.2. Cell Viability Assay

The CCK-8 method was used to determine the effects of different concentrations of Tartary buckwheat oligopeptides and H_2_O_2_ on the viability of SOL8 cells to select the optimal dose and exposure time for the oxidative stress model. Finally, the effect of Tartary buckwheat peptide on cell viability was evaluated.

#### 2.2.1. Treatment of Tartary Buckwheat Peptides

Tartary buckwheat oligopeptide (TBO) was purchased from Xi’an Haopinlai Biotechnology Company (Xi’an, China). Logarithmic growth phase SOL8 cells were seeded in 96-well plates at a density of 5 × 10^3^ cells/mL at 100 μL per well. When the cells reached 60% confluency, one week after differentiation, they were cultured in serum-free culture medium containing Tartary buckwheat oligopeptide, and the final concentrations of Tartary buckwheat oligopeptide were 0 mg/mL, 1 mg/mL, 2 mg/mL, 5 mg/mL, 10 mg/mL, 30 mg/mL, and 40 mg/mL, respectively. In addition, a control group and a serum-free culture medium group were set up. Eight compound wells were set up for each treatment, and, after 24, 48, and 72 h of culture, the absorbance of each well was detected by the CCK-8 colorimetric method at a wavelength of 450 nm1.

#### 2.2.2. Treatment of H_2_O_2_

Logarithmic growth phase SOL8 cells were seeded in 96-well plates at a density of 5 × 10^3^ cells/mL at 100 μL per well. After the cells reached 60% confluency, differentiation for one week, aspiration of the culture medium, H_2_O_2_-containing culture medium was added, the final concentration of H_2_O_2_ was 0.03 mmol/L, 0.01 mmol/L, 0.005 mmol/L, 0.002 mmol/L, 0.001 mmol/L, and 0 mmol/L, respectively. Eight double wells were set up for each treatment, and, after 1, 2, 4 h, and 24 h of treatment, the absorbance of each well was detected by the CCK-8 colorimetric method at a wavelength of 450 nm.

#### 2.2.3. Treatment of H_2_O_2_ and the Tartary Buckwheat Oligopeptide

SOL8 cells in the logarithmic growth phase were seeded in 96-well culture plates at a density of 5 × 10^3^/mL at 100 μL per well. After the cells were 60% confluent overnight, the cells were differentiated for one week, and the culture medium was aspirated. SOL8 cells were divided into a control group (CON group), H_2_O_2_ group, and pre-protection group (KB/H_2_O_2_ group). The cells in the control group and the H_2_O_2_ group were cultured in serum-free culture medium. The cells in the pre-protection group were pretreated with a final concentration of 10 mg/mL of Tartary buckwheat oligopeptide serum-free culture medium, and eight replicates were set for each treatment. After 48 h of cell culture in each group, the H_2_O_2_ group and the KB/H_2_O_2_ group were cultured with serum-free medium containing H_2_O_2_ with a final concentration of 0.01 mmol/L. The CON group was changed to serum-free culture medium for routine culture. After 1 h of treatment, the absorbance of each well was detected by the CCK-8 method at a wavelength of 450 nm.

### 2.3. Histological Examination

Histological examinations were performed to evaluate the state of cells after different stimuli and interventions. For this purpose, cells were fixed in a 10% paraformaldehyde solution and then examined for general histological features using an inverted microscope (TS100-F, EVOS M7000, Invitrogen, Waltham, MA, USA).

### 2.4. ROS Assay

Cells were stimulated by oxidative stress, and reactive oxygen species were produced and accumulated in large quantities. DHE fluorescent probing (S0064s, Beyotime, Shanghai, China) was performed using assay kits, which are used to detect intracellular reactive oxygen species, and the operation steps were carried out according to the instructions of the manufacturer. After washing the cells with PBS, 5 μmmol/L of fluorescent probe DHE was added and incubated in a cell culture incubator at 37 °C for 30 min; the PBS wash acted to remove probes that did not enter the cells, and the fluorescence intensity of each well was detected by an inverted microscope at TS100-F (EVOS M7000, Invitrogen) to indicate the ROS level. The Image J software (Version 1.54m 5 December 2024) was used to analyze the average fluorescence intensity, and the changes in ROS fluorescence intensity in integrated SOL8 cells were observed according to the average fluorescence intensity.

### 2.5. Measurement of T-AOC, Glutathione Peroxidase (CAT), MDA, and LDH Activity in SOL8 Cell

Logarithmic growth phase SOL8 cells were seeded at a density of 5 × 10^3^/mL in 96-well plates at 100 μL per well. After the cells were 60% confluent, the culture was aspirated one week after differentiation. The CON group, an H_2_O_2_ group, and a KB/H_2_O_2_ group were set up, and eight compound wells were set up for each treatment. The CON group and the H_2_O_2_ group were added with fresh serum-free culture medium for routine culture. In the KB/H_2_O_2_ group, a serum-free culture medium with a final concentration of 10 mg/mL Tartary buckwheat oligopeptide was added. After 24 h of culture, the H_2_O_2_ group and the KB/H_2_O_2_ group were replaced with fresh serum-free culture medium containing a final concentration of 0.005 mol/L H_2_O_2_. The control group was changed to fresh serum-free culture medium. After each experimental group was cultured for 1 h, the cell pellet was collected, centrifuged, and stored separately. The activities of T-AOC (BC1315, Solarbio Life Science, Beijing, China), CAT (BC0205, Solarbio Life Science, Beijing, China), LDH (BC0685, Solarbio Life Science, Beijing, China), and MDA (A003, Njjcbio, Nanjing, China) were determined according to the instructions of the kit.

### 2.6. Mitochondrial Membrane Potential Assay

The compound 5,5′,6,6′-Tetrachloro-1,1′,3,3′-tetraethylbenzimidazolylcarbocyanine iodide (JC-1) is a widely used small molecule mitochondrial membrane potential probe that relies on mitochondrial membrane potential aggregation in the mitochondria. The dye accompanies the aggregation process, and the fluorescence changes from green (530 nm) to red (590 nm). When mitochondria are depolarized, the red/green fluorescence intensity ratio decreases. After treatment, 10 μL JC-1 (G272, DOJINDO, Kumamoto, Japan) Working Solution was added and incubated in a cell culture incubator at 37 °C for 30 min, the Imaging Buffer was used to remove probes that did not enter the cells, and the fluorescence intensity of each well was detected by an inverted microscope at TS100-F (EVOS M7000, Invitrogen) to indicate the Mitochondrial Membrane Potential level.

### 2.7. Quantitative Real-Time Polymerase Chain Reaction (qRT-PCR)

The total RNA of cells was extracted in accordance with the instructions of the Total RNA Extraction Kit (R1200, Solarbio CO., Beijing, China). The RNA concentration was calculated using the ratio of OD260/280, which was detected by NanoDrop (Thermo Fisher Scientific, Waltham, MA, USA). The extracted RNA was stored at −80 °C for future use. The cDNA was obtained according to the instructions of the Reverse Transcription Kit (Solarbio CO., Beijing, China). The SYBR Green PCR kit (Solarbio CO., Beijing, China) was used for PCR amplification and quantitative analysis. The common amplification conditions were set as 95 °C 10 min, 60 °C 60 s, 72 °C 2 min, and 45 cycles. The specificity of primers was observed by the dissolution curve, and the CT values were recorded. Statistical analysis was carried out by using 2^−∆∆ct^, ΔCT = CT (target gene) − CT (GAPDH), ΔΔCT = ΔCT (target group) − ΔCt (mean value of ΔCT in control group for describing changes between groups as fold increase). All ΔΔCT converted to 2^−∆∆ct^ for subsequent statistical analysis. The oligonu cleotide primers used for PCR are as follows:

KEAP1-F: cgg gga cgc agt gat gta tg
KEAP1-R: tgt gta gct gaa ggt tcg gtt a
NRF2α-F: agc gca tct cgt tga aga ag
NRF2α-R: cog aaa tgt tga gtg tgg tg
PINK1-F, tgt atg aag cca cca tgc cc
PINK1-R: acg aca tct ggg cct ttt cc
P62-F: agt gtc cgt gtt tca cct tcc
P62-R: tgc cca gac tac gac ttg tg
HO-1-F: aag ccg aga atg ctg agt tca
HO-1-R: gcc gtg tag ata tgg tac aag ga
PGC-1-F: tgc agg cct aac tcc tcc cac
PGC-1-R: aat agg cca tcc alg gct agt cc
NRF1-F: gac cgc tgc gca tgc gct gt
NRF1-R: ggc gac gcg tac gcg aca ccc
GAPDH-F: agg tcg gtg tga acg gat ttg
GAPDH-R: ggg gtc gtt gat ggc aac a

### 2.8. Statistical Analyse

Microsoft Excel 2016 software was used to enter, organize, and calculate; SPSS 11.0 and SAS 9.4 statistical software were used to analyze the data, and an independent samples *t*-test was used to analyze the differences in cell viability, ROS, mitochondrial membrane potential, and biochemical indicators. PCR and one-way ANOVA (LSD) were used to compare the mean differences between multiple groups. We use LSD to test the significance of differences in cell viability between H_2_O_2_ and Tartary buckwheat oligopeptides concentration screening, and all values were expressed as mean ± standard deviation (X ± Σ).

## 3. Results

### 3.1. Effects of Tartary Buckwheat Oligopeptides on the Viability of SOL8 Cells

**Figure 1:**
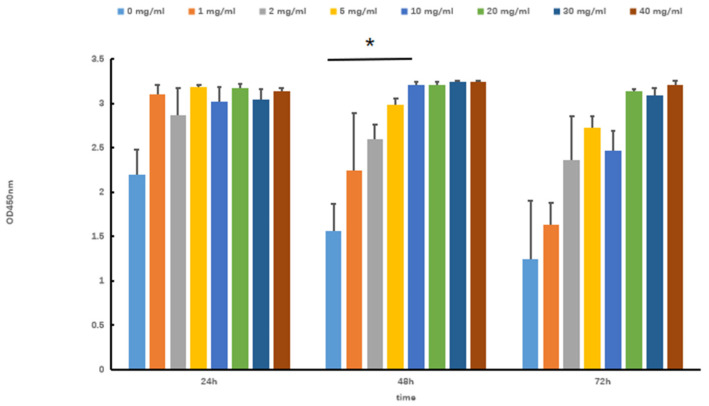
Effects of Tartary buckwheat oligopeptides on the viability of SOL8 cells (* represents *p* < 0.05).

The viability of skeletal muscle differentiation cells (SOL8) under different concentrations and times of Tartary buckwheat oligopeptides was evaluated by the CCK-8 method. As shown in Figure 1, when the concentration of Tartary buckwheat oligopeptides was 0–40 mg/mL, the cell viability showed an approximately increasing trend with the extension of intervention time, indicating that Tartary buckwheat oligopeptides did not affect the cell activity of SOL8 and had no toxic effects on cells within this concentration range. Considering the effects of concentration and time, a concentration of 10 mg/mL of Tartary buckwheat oligopeptides was selected for continuous experiments lasting 48 h (compare with 0 mg/mL, *p* < 0.05).

### 3.2. Effects of H_2_O_2_ on Cell Viability

**Figure 2:**
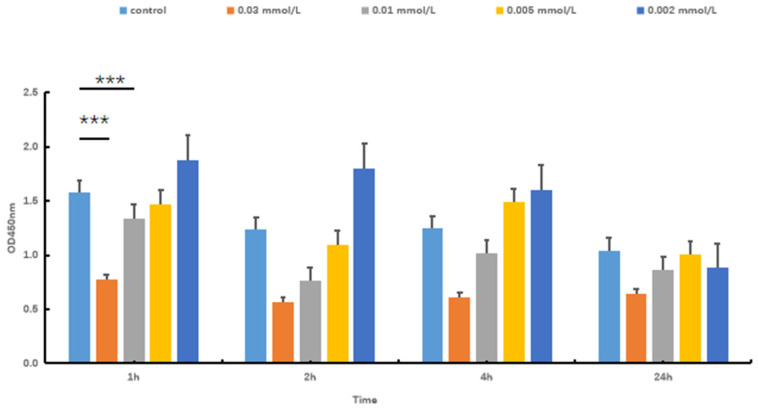
Effects of H_2_O_2_ concentration and time on cell viability (*** represents *p* < 0.001).

Hydrogen peroxide (H_2_O_2_) is capable of directly oxidizing lipids and proteins. Additionally, it can permeate the cell membrane via chemical reactions, thereby accumulating free radicals. This process ultimately results in cellular damage. To establish a model of oxidative stress injury in SOL8, different concentrations of H_2_O_2_ were used to evaluate the cytotoxicity to select an appropriate concentration. The results showed that H_2_O_2_ is capable of decreasing SOL8 cell viability in a dose-dependent manner (Figure 2). The CCK-8 value of the 0.01 mol/L H_2_O_2_ group was significantly lower than that of the control group after 1 h of cell damage (*p* < 0.001), and the cell survival rate was significantly higher than that of the 0.03 mol/L H_2_O_2_ group. Considering the intervention dose and time, an H_2_O_2_ concentration of 0.01 mol/L for 1 h was used to establish a model of oxidative stress injury in SOL8.

### 3.3. Effects of Tartary Buckwheat Oligopeptides on SOL8 Cell Viability in Oxidative Stress

**Figure 3:**
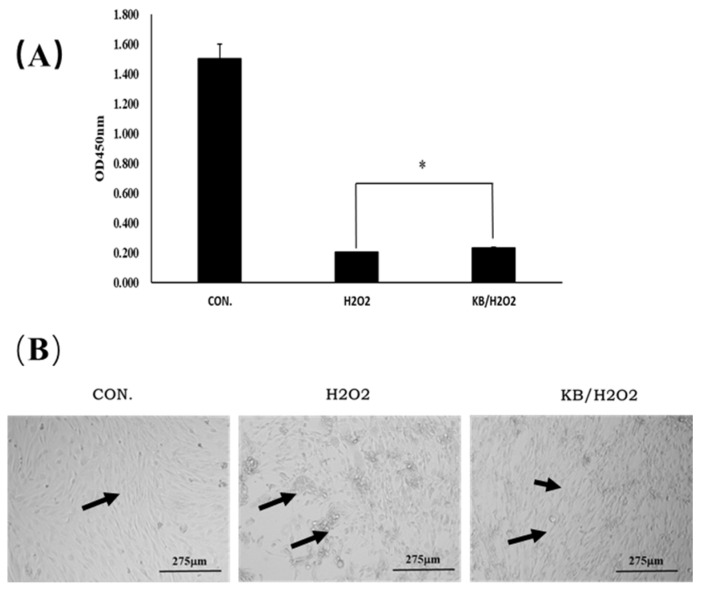
Effects of Tartary buckwheat oligopeptides on the viability of SOL8 cells under oxidative stress. (**A**) Represents viability of SOL8 cells; (**B**) micrograph of SOL8 cell growth status; the arrows represent the differentiated SOL8 cells (* represents *p* < 0.05).

As shown in Figure 3A, the cell proliferation activity of the H_2_O_2_ group was decreased after H_2_O_2_ stimulation; Tartary buckwheat oligopeptides significantly enhanced SOL8 cell viability in H_2_O_2_-insulted cells (*p* < 0.05). The same trend can be seen from the microscopic images (Figure 3B); SOL8 cells lost the complete morphology of differentiated skeletal muscle under the stimulation of hydrogen peroxide, as shown by the arrows in Figure 3B. The intervention of Tartary buckwheat oligopeptides partly maintained the morphological integrity of the cells. These results indicated that Tartary buckwheat oligopeptides could enhance the cell viability of SOL8 cells that have been injured by H_2_O_2_ and reduce damage to SOL8 cells.

### 3.4. Effects of Tartary Buckwheat Peptides on the Production of ROS in Oxidative Stress-Injured SOL8 Cells

**Figure 4:**
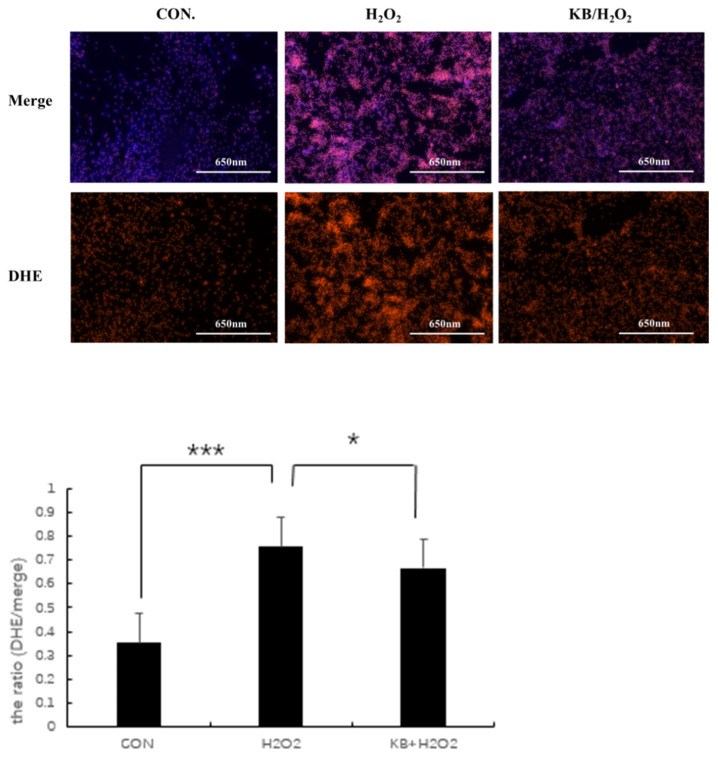
Effects of Tartary buckwheat peptides on the ROS Levels in SOL8 cells under oxidative stress. (*** represents *p* < 0.001, * represents *p* < 0.05).

Dihydroethidium (DHE) is the most commonly used fluorescent probe for detecting intracellular superoxide anion levels. DHE itself emits blue fluorescence, and, when DHE reacts with superoxide anions in cells, the generated ethidium binds to RNA or DNA to produce red fluorescence. The stronger the red fluorescence, the higher the level of superoxide anions in the cells. In the present study, excessive ROS was generated in H_2_O_2_-injured cells, as shown in Figure 4, compared with the intracellular fluorescence intensity of the CON group; the red fluorescence in the H_2_O_2_ group significantly increased, and the relative fluorescence value of ROS was extremely significant (*p* < 0.001). With the addition of Tartary buckwheat peptides, the intracellular ROS content was markedly reduced compared with the H_2_O_2_ group (*p* < 0.05), approaching the level of the control group, and the relative fluorescence value of ROS in the KB/H_2_O_2_ group was lower than that in the H_2_O_2_ group. These results indicated that Tartary buckwheat peptides exerted an antioxidant effect on the oxidative stress-injured SOL8 cells.

### 3.5. Effects of Tartary Buckwheat Peptides on the Levels of MDA, SOD, and CAT in SOL8 Cells Under Oxidative Stress

**Figure 5:**
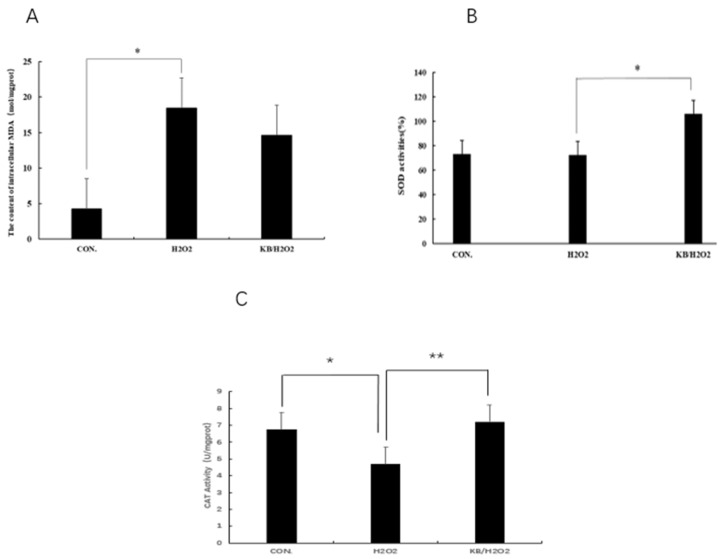
Effects of Tartary buckwheat peptides on the levels of MDA, SOD, and CAT in SOL8 cells under oxidative stress. ((**A**): MDA content of SOL8; (**B**): SOD content of SOL8; (**C**): CAT content of SOL8; ** represents *p* < 0.01, * represents *p* < 0.05).

ROS can easily react with lipids through double bonds, leading to cell membrane degradation and the production of lipid oxidation products such as MDA. Therefore, the content of MDA can reflect the degree of oxidative damage in cells. As shown in Figure 5A, the MDA content in the H_2_O_2_ group was significantly higher than that in the CON group (*p* < 0.05), and the MDA content in the KB/H_2_O_2_ group was 11.5% lower than that in the H_2_O_2_ group. Tartary buckwheat oligopeptides have a protective effect on the oxidative stress of SOL8 cells, possibly by increasing the activity of antioxidant enzymes and reducing the level of lipid oxidation in cells, which is conducive to maintaining cellular redox homeostasis. Superoxide dismutase (SOD) is a widely existing antioxidant enzyme in living organisms and plays a key role in the balance of oxidation and antioxidant in the body. After intervention with H_2_O_2_, the activity of SOD in cells was decreased, and the SOD activity in the KB/H_2_O_2_ group was significantly higher than that in the H_2_O_2_ group (*p* < 0.05), which can inhibit inflammation and apoptosis mediated by intracellular signals (Figure 5B). Catalase (CAT) is an enzyme that can decompose hydrogen peroxide into water and oxygen, protecting cell components such as proteins and DNA from hydrogen peroxide attack and, thus, protecting cells from damage. The activity of CAT (Figure 5C) in the H_2_O_2_ group was significantly lower than that in the CON group (*p* < 0.05), and the activity of CAT in the KB/H_2_O_2_ group was significantly higher than that in the H_2_O_2_ group (*p* < 0.01). CAT and SOD, as antioxidant enzymes in the antioxidant system within living organisms, both convert active substances into harmless substances, thereby protecting cells from oxidative damage.

### 3.6. Effect of Tartary Buckwheat Peptides on Mitochondrial Function in SOL8

**Figure 6:**
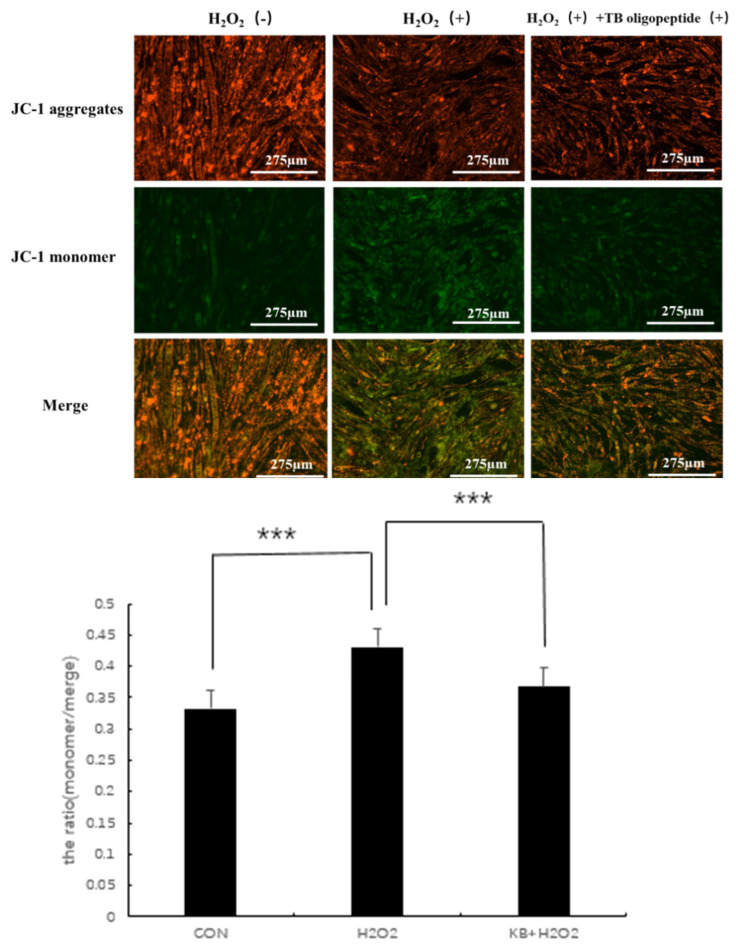
Effects of Tartary buckwheat peptides on mitochondrial membrane potential (*** represents *p* < 0.001).

Mitochondria in cells are one of the important organelles in the body. As the main site of aerobic respiration in cells, they are commonly used in the study of early cellular oxidative stress and apoptosis. Mitochondrial activity is associated with aging and diseases, and changes in mitochondrial membrane potential are often used as an important detection index. JC-1 is a membrane-permeable dye widely used in flow cytometry and fluorescence microscopy to measure mitochondrial membrane potential. When the mitochondria are normal and the membrane potential difference remains unchanged, JC-1 will aggregate and emit red fluorescence. When the membrane potential decreases, JC-1 will emit green fluorescence as a monomer, and the ratio of the two can be used as an indicator to detect the state of mitochondria. As shown in Figure 6, compared with the CON group, the ratio (the ratio of green fluorescence to total fluorescence intensity) in the H_2_O_2_ group significantly increased (*p* < 0.001), and the mitochondrial membrane potential in the KB/H_2_O_2_ group tended to return to the level of the CON group, with the ratio (the ratio of green fluorescence to total fluorescence intensity) significantly lower than that in the H_2_O_2_ group (*p* < 0.001).

### 3.7. Expression of Mitophagy-Related Genes in SOL8 Cells

**Figure 7:**
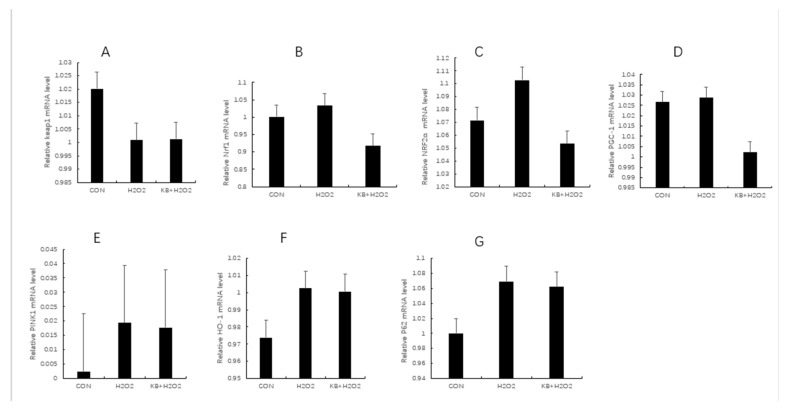
The mRNA levels of mitophagy-related genes in SOL8 cells. (The labels (**A**–**G**) correspond to KEAP1, NRF1, NRF2α, PGC-1, PINK, HO-1, and P62, respectively. KEAP1: Kelch-like ECH-associated protein 1; NRF1: nuclear respiratory factor 1; NRF2α: Nuclear Factor erythroid 2-Related Factor 2; PGC-1: Peroxisome proliferator activated receptor-γ coactivator-1; PINK: PTEN induced putative kinase 1; HO-1: Heme Oxygenase-1; P62: prostacyclin).

As shown in Figure 7A–G, compared with the CON group and the KB/H_2_O_2_ group, the mRNA expression of KEAP1 in the H_2_O_2_ group was decreased. Since the activity of KEAP1 was resisted, NRF2α was no longer ubiquitinated and degraded, thus accumulating and transferring to the nucleus to activate the expression of antioxidant genes. Therefore, the mRNA expression of NRF1 and NRF2α in the H_2_O_2_ group was higher than that in the CON group and the KB/H_2_O_2_ group. HO-1, as a downstream target gene of NRF2, had higher mRNA expression in the H_2_O_2_ group than in the CON group and the KB/H_2_O_2_ group. The metabolites of HO-1 can further regulate the expression of PGC-1 through stimulating signaling pathways; therefore, as shown in Figure 7D,F, the mRNA expression of HO-1 and PGC-1 in the H_2_O_2_ group was higher than that in the CON group and KB/H_2_O_2_ group. P62 binds to KEAP1 to further promote the release and nuclear translocation of NRF2. The mRNA expression of P62 in the H_2_O_2_ group was higher than that in the control group and the intervention group (Figure 7G). As depicted in Figure 7E, H_2_O_2_ in the PINK-1 group increased, while that in the KB/H_2_O_2_ group decreased. However, these changes did not reach statistical significance.

## 4. Discussion

During cellular metabolism, a moderate level of reactive oxygen species (ROS) serves as a signaling molecule that helps maintain intracellular homeostasis. We observed a significant reduction in ROS production after 48 h of pre-treatment with Tartary buckwheat oligopeptides. This indicates that Tartary buckwheat peptides effectively scavenge intracellular ROS in SOL8 cells subjected to H_2_O_2_-induced oxidative stress. While ROS generation is an inevitable byproduct of cellular oxidative metabolism, excessive accumulation of ROS can disrupt normal cellular physiological functions. When ROS levels become too high, free radical metabolism becomes dysregulated, leading to oxidative damage to cellular macromolecules such as DNA and proteins. In our study, the relative content of ROS in the hydrogen peroxide group increased significantly, and the relative content of ROS in SOL8 cells in the intervention group decreased significantly, which confirmed that Tartary buckwheat oligopeptide could protect cells from hydrogen peroxide attack, indicating that Tartary buckwheat oligopeptide can reduce the degree of cell oxidative damage by removing intracellular ROS.

Mitochondria are the powerhouses of the cell, primarily responsible for cellular energy metabolism, generating the energy (ATP) required by the cell through the process of oxidative phosphorylation [14]. However, during normal physiological functioning or when damaged, mitochondria produce certain by-products, the most important of which are reactive oxygen species (ROS). When mitochondria are damaged [15], their normal functions are compromised, leading to the overproduction of ROS. Excessive release of ROS from damaged mitochondria is an important link in causing intracellular oxidative stress, which can lead to cell damage. This phenomenon is specifically reflected by the mitochondrial potential membrane changes, i.e., the red aggregates in cells exposed to hydrogen peroxide gradually disappear, and the green fluorescence of the monomer diffuses in the cytoplasm compared to the mitochondrial potential membrane of SOL8 differentiated cells protected by Tartary buckwheat oligopeptide. Numerous reports [16,17,18] have confirmed that bioactive peptides can protect mitochondria from oxidative stress-induced damage. For instance, in a mouse model of gouty arthritis, alginate oligosaccharide (AOS3) reduces oxidative stress and ROS production, restores mitochondrial function, and activates the Nrf2 pathway to promote the expression of antioxidant genes [19]. The SS-31 peptide, a widely studied mitochondria-targeted antioxidant peptide, can penetrate the mitochondrial membrane, reduce ROS production, inhibit lipid peroxidation, and protect cells from oxidative damage. For example, SS-31 has demonstrated significant protective effects in various disease models, including myocardial infarction, renal fibrosis, and ischemia-reperfusion injury [20]. This is consistent with the results of mitophagy experiments, which indicate a clearing mechanism in which malfunctioning mitochondria, due to causes such as oxidative stress and DNA damage, are wrapped into autophagosomes through autophagy, which are then degraded after fusion with lysosomes. In the experiment, after phagosome–lysosomal fusion, compared with the blank group and the intervention group, the stain showed stronger fluorescence in an acidic environment after the pH decreased.

Under oxidative stress, the mRNA expression of multiple genes undergoes changes to enhance the cellular antioxidant defense mechanisms. Some studies have shown that changes in the expression levels associated with the Keap1 pathway can enhance the cellular antioxidant capacity by altering cellular metabolic pathways. In the articles published by Prashant Deshmukh [21], Bae SH [22], and Bryan HK [23], the expression of Keap1 and NRF2α is closely related to the levels of ROS in cells. Initially, the reactive oxygen species (ROS) generated by oxidative stress modify the cysteine residues of KEAP1, leading to its inactivation and the inability to suppress the activity of NRF2α. In this experiment, the expression of KEAP1 in the hydrogen peroxide group was higher than that in the control and intervention groups, further proving that, after 48 h of intervention with Tartary buckwheat oligopeptides, under the same stimulation conditions, the ROS content in the intervention group cells was lower than that in the hydrogen peroxide group cells.

When NRF2α accumulates in the cytoplasm and then translocates to the nucleus, it binds to the antioxidant response element (ARE) in the nucleus, promoting the mRNA expression of antioxidant genes such as HO-1 and NQO1 [13,24]. At the same time, the mRNA expression of P62 is also upregulated. The P62 protein can bind to KEAP1, further releasing NRF2α and enhancing its nuclear translocation [25], which is in line with the results of this experiment, indicating that Tartary buckwheat peptides can influence mRNA expression to protect cells from oxidative stress. In addition, the mRNA expression of PGC-1α increases, promoting mitochondrial biogenesis and enhancing the cell’s antioxidant capacity [26,27]. The mRNA expression of PINK1 is upregulated, which aids in mitophagy, which acts to remove damaged mitochondria [28]. The mRNA expression of NRF1 is also upregulated, similar to NRF2, in regulating the expression of antioxidant genes. The aforementioned results are consistent with the majority of previous reports on oxidative stress [29,30,31,32]. These changes in mRNA expression of genes work together to help cells cope with oxidative stress and maintain cellular homeostasis.

In our previous study [33], we analyzed the 17 kinds of amino acids of the Tartary buckwheat oligopeptides in the hydrolysate (tryptophan not tested). The total amino acid content of Tartary wheat oligopeptides was 82.2 g/100 g, and it contained seven kinds of essential amino acids (Val, Iie, Leu, Phe, Met, Thr, Lys), among which the contents of glutamic acid, proline, leucine, and serine were higher. The mass fraction of five kinds of amino acids (glutamic acid, leucine, valine, alanine, and lysine) with antioxidant activity was about 46.28%. The content of alanine, glycine, and valine was 15.02%, suggesting that the intake of Tartary buckwheat oligopeptide may improve exercise ability. Glutamate and aspartate have the properties of reducing fatigue. Tartary buckwheat oligopeptide has 34.75% glutamic acid and 2.63% aspartic acid, which suggests that it probably has a certain anti-fatigue effect. Moreover, we used GSH as a positive control to demonstrate in vitro that Tartary buckwheat oligopeptides have the ability to scavenge DPPH and OH- free radicals. The study design is mainly centered on SOL8 cells, with the primary objective of investigating the role of a novel cell signaling pathway in cell apoptosis. The experimental design is aimed at verifying this mechanistic hypothesis rather than directly comparing the effects of different drugs. Therefore, at this stage, we have not included a positive control group. Before proceeding to subsequent animal studies, we will add a positive control group to lay the groundwork for further in vivo validation of the related research hypothesis. If classic antioxidants (such as vitamin *C* and *N*-acetylcysteine) were included as control groups, the protective effects of Tartary buckwheat oligopeptides on cells would be more fully demonstrated and assessed. The experiment will further analyze the structure of Tartary buckwheat oligopeptides and establish in vivo studies based on animal models to analyze the bioavailability and metabolites of the active peptides. Given the antioxidant properties of Tartary buckwheat oligopeptides, they will have promising applications in the fields of food and cosmetics.

## 5. Conclusions

In summary, the current study revealed that Tartary buckwheat oligopeptides prevented the accumulation of ROS in SOL8, activated the cellular antioxidant defense system, and decreased the accumulation of MDA, protecting intracellular LDH levels. This indicates that Tartary buckwheat oligopeptides on cells not only safeguard endogenous antioxidant enzymes from damage but may also enhance their ability to resist oxidative stress damage. Additionally, the direct effect of Tartary buckwheat oligopeptides on mitochondria brought about a reduction in oxidative stress, regulation of mitochondrial function, such as MMP, and attenuation of apoptosis. Based on these results, Tartary buckwheat oligopeptides could be considered as a potential treatment option for H_2_O_2_-induced oxidative stress damage and apoptosis in SOL8 cells, providing theoretical guidance for their further application in functional products.

## Data Availability

The original contributions presented in this study are included in the article; further inquiries can be directed to the corresponding author.
